# Influence of Discontinuation of Cardiac Rehabilitation in Elderly Outpatients Due to the COVID-19 Pandemic

**DOI:** 10.3390/jcdd9060194

**Published:** 2022-06-18

**Authors:** Noriyuki Mouri, Yasunori Suematsu, Yuiko Yano, Kai Morita, Miki Shirosaki, Masaomi Fujita, Takuro Matsuda, Sakiko Matsuo, Rie Tazawa, Kanta Fujimi, Shin-ichiro Miura

**Affiliations:** 1 Department of Cardiology, Fukuoka University Hospital, Fukuoka 814-0180, Japan; momouri0327@gmail.com (N.M.); yuicom0109@gmail.com (Y.Y.); kurisuchan0924@gmail.com (K.M.); kanta2345@yahoo.co.jp (K.F.); 2 Division of Rehabilitation, Fukuoka University Hospital, Fukuoka 814-0180, Japan; jovianorbit@yahoo.co.jp (M.S.); omiomi1973@gmail.com (M.F.); gd050010@yahoo.co.jp (T.M.); 3 Division of Nutrition, Fukuoka University Hospital, Fukuoka 814-0180, Japan; matsuo1021@fukuoka-u.ac.jp (S.M.); tazawa0220@fukuoka-u.ac.jp (R.T.); 4 Department of Cardiology, Fukuoka University Nishijin Hospital, Fukuoka 814-0180, Japan

**Keywords:** standing balance, muscle mass, cardiac rehabilitation, COVID-19

## Abstract

Background: The coronavirus disease 2019 (COVID-19) pandemic has restricted people’s activities and necessitated the discontinuation of cardiac rehabilitation (CR) programs for outpatients. In our hospital, CR for outpatients had to be discontinued for 3 months. We investigated the influence of this discontinuation of CR on physical activity, body composition, and dietary intake in cardiovascular outpatients. Method: Seventy-eight outpatients who restarted CR were investigated. We measured body composition, balance test, stage of locomotive syndrome, and food frequency questionnaire (FFQ) results at restart and 3 months later. We also investigated the results of examination that were obtained before discontinuation. Results: With regard to baseline characteristics, the percentage of male was 62.7% (*n* = 49), and average age and body mass index were 74.1 ± 8.5 years and 24.9 ± 7.0 kg/m^2^, respectively. Stage of locomotive syndrome and the results of FFQ did not change significantly. The one-leg standing time with eyes open test significantly worsened at restart (*p* < 0.001) and significantly improved 3 months later (*p* = 0.007). With regard to body composition, all limb muscle masses were decreased at restart and decreased even further 3 months later. Conclusions: Discontinuation of CR influenced standing balance and limb muscle mass. While the restart of CR may improve a patient’s balance, more time is required for additional daily physical activities. The recent pandemic-related interruption of CR should inspire the development of alternatives that could ensure the continuity of CR in a future crisis.

## 1. Introduction

A novel severe acute respiratory syndrome coronavirus (SARS-Cov-2) called coronavirus disease 2019 (COVID-19) was first identified in China in December 2019. COVID-19 is highly infective and the number of infected people increased rapidly [1]. Since early 2020, social isolation has been required to help prevent the spread of COVID-19, and this has restricted participation in physical activities [2]. The first infected person in Japan was identified in January 2020 and Japan experienced six waves of COVID-19 to March 2022. During the pandemic, people have limited and/or modified activities including sightseeing, leisure, eating out, student’s school life, working, and even exercising at the gym. At Fukuoka University Hospital, Fukuoka, Japan, we discontinued cardiac rehabilitation (CR) for outpatients from April 2020 to June 2020 during the COVID-19 pandemic. This discontinuation of CR further limited the physical activities of outpatients with cardiovascular diseases (CVD) who were attending CR.

CR improves the CVD prognosis of CVD, quality of life (QOL), and activities of daily living in CVD outpatients. Exercise-based CR decreases all-cause mortality and hospitalization, and improves QOL [3]. Discontinuation of CR for CVD outpatients due to the COVID-19 pandemic might reduce a patient’s physical activities. The COVID-19 pandemic also affects dietary behaviors [4]. In this study, we investigated the influence of the discontinuation of CR due to the COVID-19 pandemic on physical activity, body composition, and dietary habits in CVD outpatients.

## 2. Materials and Methods

### 2.1. Study Design

At Fukuoka University Hospital, Fukuoka, Japan, we discontinued CR for outpatients from April 2020 to June 2020 due to the COVID-19 pandemic. Seventy-eight CVD outpatients at the CR center of Fukuoka University Hospital were enrolled in this study. We measured body composition, balance test, stage of locomotive syndrome, and food frequency questionnaire (FFQ) at the restart of CR and after 3 months from restart. We investigated these examinations form April 2019 to March 2020 from the data, before discontinuation. Although we also intended to collect the results of examinations before discontinuation, the FFQ data from only 10 patients were actually collected because the decision to discontinue CR at our hospital was made quite suddenly.

The study was performed in accordance with the Declaration of Helsinki and the ethical standards of the Independent Review Board of Fukuoka University. The protocol was approved by the Independent Review Board of Fukuoka University (U20-785). Informed consent was obtained in the form of opt-out on a web-site.

### 2.2. CR Program

Outpatients come to the hospital 1–2 times a week for 60 min each time. At each session, the patient’s vital signs are first checked and the medical staff ask if they have any abnormalities to date. After 5 min of warm-up exercises, the participants perform aerobic exercise for 30 min and stretching for 15 min. Finally, their vital signs are checked again to see if there are any abnormalities. Patients are also evaluated with respect to nutrition, medical examination, education, lifestyle guidance and psychological support, in addition to exercise, by our medical CR care team at starting CR, 5 months later, and when needed. The team is a multidisciplinary collaboration, and the team members include a physician, nurse, psychologist, pharmacist, nutritionist and exercise instructor.

### 2.3. Patient Characteristics before Restart CR

Age, gender, body mass index, current smoker, hypertension, diabetes mellitus, dyslipidemia, chronic kidney disease, and chronic obstructive pulmonary disease were investigated. As underlying CVD, ischemic heart disease, heart failure, cardiomyopathy, macrovascular disease, and peripheral artery disease were investigated. Some patients had multiple CVD. The period of participation in the CR program, the number of times the subject participated in the CR program per month, serum brain natriuretic peptide, and left ventricular ejection fraction by echocardiography before COVID-19 pandemic were also investigated.

### 2.4. Body Composition

Body composition was checked by an MC-190 (TANITA, Itabashi-Ku, Tokyo, Japan). Body weight, body mass index, body fat mass, percent body fat, body muscle mass, estimated bone mass, basal metabolic rate, trunk fat mass, and muscle mass at the trunk, right arm, left arm, right leg, and left leg were measured.

### 2.5. Staging of Locomotive Syndrome

Locomotive syndrome is a condition wherein mobility, as manifested by activities such as sit-to-stand or gait, is impaired due to deficits in locomotive organ function [5]. Patients performed the stand-up test, 2-step test, and a 25-question geriatric locomotive function scale, and the stage of locomotive syndrome was calculated according to the instructions. [6].

### 2.6. One-Leg Standing Time with Eyes Open Test

The one-leg standing time with eyes open test was performed with hands on waist. We recorded the time until the subject lost balance, moved their hands from their waist, or touched the ground with their raised foot [7]. Patients also performed the both-legs test and we measured the time, with a maximum score of 60 s.

### 2.7. FFQ

The FFQ Ver.5 (Kenpakusha, Tokyo, Japan) asks participants to report their consumption of several types of food over the previous 1 or 2 months. Participants answer the amount per time and the frequency per week of 29 food items and 10 cooking methods, including grains, meat, fish, vegetables, fruit, sweet, drink, oil, and salt. Participants were also asked questions regarding their daily activities, such as hours of sleep, 3-grade living activities, and exercise per week. After analysis by attached software, total calorie intake, calorie intake for proteins, carbohydrates, and fats, intake of salt, calorie expenditure, hours of living activities and exercise per day were recorded.

### 2.8. Statistical Analysis

All data analyses were performed using SAS (Statistical Analysis System) Software Package (Ver. 9.4, SAS Institute Inc., Cary, NC, USA) at Fukuoka University (Fukuoka, Japan). Continuous variables with a normal distribution were expressed as mean ± standard deviation, continuous variables with a non-normal distribution were expressed as median (interquartile range) and categorical variables were expressed as number, (%). We investigated the changes in the variables at restart of CR and after 3 months of CR. The paired t-test was performed for changes in variables that showed a normal distribution. The Wilcoxon signed-rank test was performed for changes in variables that showed a non-normal distribution. Differences were evaluated by a two-sided test with an alpha level of 0.05. A value of *p* < 0.05 was considered significant.

## 3. Results

### 3.1. Patient Characteristics before Discontinuation

Patients had initially started CR 4.2 ± 2.5 years before discontinuation and had participated in a CR program 4 (3–7) times per month from January 2020 to March 2020. All patients were in the maintenance period of CR and their clinical condition had been stable. Age and percentages of male and current smoking were 74.1 ± 8.5 years, 62.8% (*n* = 49), and 9.1% (*n* = 6), respectively (Table 1). With regard to underlying heart disease, the percentages of patients with ischemic heart disease, heart failure, cardiomyopathy, macrovascular disease, and peripheral artery disease were 57.7% (*n* = 45), 51.3% (*n* = 40), 10.3% (*n* = 8), 15.4% (*n* = 12), and 10.3% (*n* = 8), respectively. The average serum brain natriuretic peptide and left ventricular ejection fraction were 79.7 (49–198) pg/mL and 60.9 ± 12.5%, respectively. The percentage of patients with heart failure with preserved ejection fraction in all HF was 74%. After restart of CR, patients participated in the program 4 (3–6) times per month from July 2020 to September 2020.

### 3.2. Changes in the FFQ Findings at Restart of CR and after 3 Months

We investigated the frequency of food intake in patients before discontinuation, at restart of CR and after 3 months by the FFQ (Table 2). Only ten patients completed the FFQ from April 2019 to March 2020. The total calorie intake and the calorie percentages of protein, fat, and carbohydrate were not significantly different at restart of CR or after 3 months. Salt intake at restart of CR increased from 8.77 ± 3.00 g/day to 9.35 (7.0–12.0) g/day, but this difference was not significant. The low rate of completing the FFQ before discontinuation of CR could explain this lack of a significant change. FFQ can assign calorie expenditure due to exercise, active living activities, normal living activities, and rest living activities. While there were no significant changes, calorie expenditure by active and normal daily activities tended to increase at restart CR and after 3 months. These changes would be affected by the discontinuation of CR due to COVID-19 pandemic.

### 3.3. Changes in Physical Activity at Restart of CR and after 3 Months

**Figure 1:**
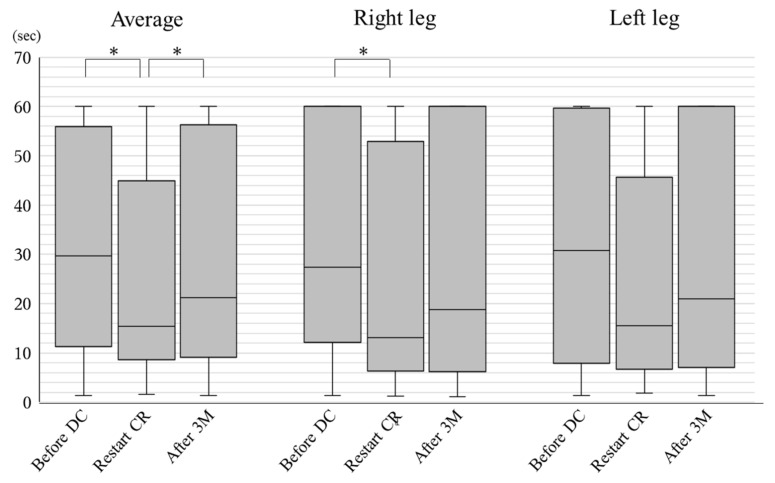
Changes in standing balance before DC, at restart of CR and after 3 months. The one-leg standing time with eyes open test was performed. The times for the right leg, left leg, and their average before discontinuation (DC), at restart cardiac rehabilitation (CR), and after 3 months (M) CR are shown. * indicates *p* < 0.05.

The stage of locomotive syndrome before discontinuation was 1 (0–2) (*n* = 49). There were no significant differences (before discontinuation vs. at restart CR, *p* = 1.0, at restart CR vs. after 3 months, *p* = 0.90, and before discontinuation vs. after 3 months, *p* = 0.18). The results of the one-leg standing time with eyes open test are shown in Figure 1. The average time was 29.7 (11.7–55.1) sec before discontinuation (*n* = 48), 15.4 (8.6–45.0) sec at restart of CR (*n* = 43), and 21.2 (9.1–54.6) sec after 3 months (*n* = 62). The time significantly decreased from before discontinuation to restart of CR (*p* < 0.001) and significantly increased from restart of CR to after 3 months (*p* = 0.007).

### 3.4. Change in Body Composition at Restart of CR and after 3 Months

**Figure 2:**
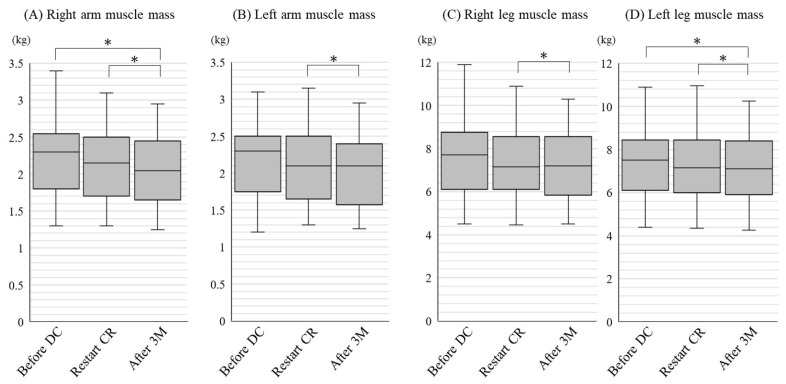
Changes in limb muscle mass before DC, at restart of CR and after 3 months. Limb muscle mass was investigated by a body composition analyzer. (**A**) right arm, (**B**) left arm, (**C**) right leg, (**D**) left leg muscle mass at each timing before discontinuation (DC), at restart of cardiac rehabilitation (CR), and after 3 months (M) of CR are shown. * indicates *p* < 0.05.

Table 3 shows the changes in body composition. From 63.0 (54.3–67.5) kg before discontinuation, body weight decreased to 60.7 ± 10.8 kg at restart of CR (*p* = 0.94) and to 60.2 ± 10.6 kg after 3 months (*p* = 0.49). Body fat mass was similar in all of the time periods. The decrease in body weight was believed to be due to reductions in estimated bone mass and body muscle mass. From 2.50 (2.20–2.70) kg before discontinuation, estimated bone mass decreased to 2.35 (2.15–2.65) kg at restart of CR (*p* < 0.001) and 2.35 (2.10–2.60) kg after 3 months (*p* = 0.009). From 44.5 (37.0–48.6) kg before discontinuation, body muscle mass decreased to 41.7 ± 7.2 kg at restart of CR (*p* = 0.41) and to 41.6 ± 7.2 kg after 3 months (*p* = 0.20). Especially with regard to the limb muscle mass, for all of the limbs, muscle mass decreased at restart of CR and decreased further after 3 months (Figure 2). Although there were no significant changes in trunk muscle mass between before DC, restart of CR and after 3 months in all patients, the trunk muscle mass significantly increased to 23.4 ± 3.6 kg after 3 months CR from 23.1 ± 3.5 kg at restart of CR in non-diabetic patients (*p* = 0.04), but not in diabetic patients (after 3 months CR: 22.9 ± 3.4 kg, vs. at restart of CR: 22.9 ± 3.4 kg, *p* = 0.24).

## 4. Discussion

In this study, we investigated the influence of discontinuation of CR for outpatients due to the COVID-19 pandemic. Although patients showed a worsening of their standing balance during discontinuation, this recovered after 3 months of CR. Muscle mass, especially limb muscle mass, decreased during discontinuation, and decreased further after 3 months of CR. It may be difficult for limb muscle mass to recover with short term CR, and additional physical activity in daily living may be needed, in addition to CR.

First, in this study, muscle mass decreased during discontinuation of CR, and decreased further after 3 months of CR. In our hospital, the CR program includes aerobic exercise with an exercise load that is appropriate for each patient for 30 min and resistance training in a group for 20 min. During discontinuation, we provided exercise videos through social media and instructed CR outpatients to keep performing home-based exercise. Nonetheless, their standing balance and limb muscle mass worsened. It was difficult for patients to maintain their standing balance and muscle mass during the COVID-19 pandemic. Japanese guidelines on rehabilitation in patients with CVD recommended that patients perform aerobic exercise under the anaerobic threshold for 30–60 min a day, at least 5 days per week [8]. Both before discontinuation and after restart of CR, the total exercise time was not enough because they performed only CR. Our outpatients with CR had already established some exercise, dietary and lifestyle habits because they had performed CR for 4.2 ± 2.5 years. Nevertheless, the finding that a temporary discontinuation of CR for outpatients increased muscle weakness is very important. Under normal non-pandemic conditions, if CR had simply temporarily stopped, patients would have been able to go out and engage in various activities without developing muscle weakness. However, due to the need for social isolation during the COVID-19 pandemic in Japan, daily activities became limited and patients experienced anxiety, even after restart of CR. Under these conditions, we believe it was difficult for outpatients to maintain their desired lifestyle.

In fact, Yamada et al. reported that, among older adults, the total physical activity time in April 2020 significantly decreased compared to that in January 2020 due to the COVID-19 pandemic, which suggests that a higher incidence of disability can be expected in the near future in this group [9]. The total physical activity time during the first, second, and third waves of the COVID-19 pandemic decreased from the pre-pandemic level by 33.3%, 28.3%, and 40.0%, respectively [10]. Home-based CR would be suitable under these conditions. Home-based CR has been shown to have effects similar those of center-based CR on quality of life among ischemic heart disease patients [11]. Home-based mobile-guided CR has been shown to be safe and effective for improving peak oxygen uptake among elderly patients in Europe [12]. Supervised exercise programs delivered online in addition to education provided effective and accessible CR during the COVID-19 pandemic [13]. Mukaino et al. established the feasibility of a telerehabilitation system for patients who are quarantined due to COVID-19 by combining existing commercial devices and computer applications [14]. In Japan, a multicenter study on the efficacy of remote technology in CR is ongoing. While a system of home-based CR has not yet been established in Japan, we expect that there may be an even greater need for such a system in the future.

In addition, the recovery of muscle mass could be difficult with only 3 months of CR. The effect of CR on muscle mass varies according to the patient background and the duration of CR. It has been reported that 6 months of CR increased muscle after coronary artery bypass grafting among non-diabetic patients, but not diabetic patients [15]. In this study, the trunk muscle mass significantly increased after 3 months of CR in non-diabetic patients, but not in diabetic patients. The reason why diabetes accelerates the aging of skeletal muscle could involve oxidative stress, chronic inflammation, insulin resistance, mitochondrial dysfunction, and the accumulation of advanced glycation end-products [16]. If non-diabetic patients were to perform CR for longer in each session and/or increase the number of CR sessions per week, other muscle mass might increase. The estimated bone mass significantly decreased at restart of CR and decreased even further after 3 months. Exercise is an important element to improve bone mineral density [17]. Discontinuation of CR and social isolation due to the COVID-19 pandemic might have contributed to a decrease in bone mass.

Second, although patients showed a worsening of their standing balance during discontinuation, 3 months of CR recovered their balance without a recovery of muscle mass. The one-leg standing time with eyes open test is one of the major balance tests and is widely used in elderly patients [18]. Postural control consists of stability and orientation [19]. Stability is the ability to maintain one’s center of mass. Orientation is the ability to maintain the association of multiple body segments and the association between the body and the environment. Stability and orientation are associated with the skeletal muscular system, sensory system, and central nervous system [20]. Short-term CR might help to recover these systems. In addition, although it has been reported that muscle strength rather than muscle mass is associated with standing balance in elderly outpatients, [21] we did not measure muscle strength. It is possible that short-term CR might help recover muscle strength.

Third, the change in dietary responses during the COVID-19 pandemic varied among individuals [4]. Some reports have shown that healthy behaviors increased during the COVID-19 pandemic [22,23]. On the other hand, Visser et al. showed that the COVID-19 pandemic had a negative impact on the nutritional behavior of many older adults, which may increase their risk of malnutrition, frailty, sarcopenia and disability [24]. Diet was correlated with frailty in older adults living in the community during the period of restrictions on outings due to COVID-19 [25]. In this study, total calorie intake and the calorie percentages of protein, fat, and carbohydrate did not change. Salt intake at restart of CR increased from 8.77 ± 3.00 g/day before discontinuation to 9.35 (7.0–12.0) g/day, but this difference was not significant. Further studies will be needed to elucidate the influence of the COVID-19 pandemic on food intake by outpatients with CR.

This study has several limitations. First, the study was small, and the data before discontinuation was limited. Especially, age was high and BMI was low in this study compared to other studies [26,27]. Regional difference regarding baseline characteristics might need to be considered. Second, there was a difference in the number of patients at each time, because some of the data were missing. However, since we used the Paired t-test or Wilcoxon signed-rank test to evaluate the significance of changes in variables, we do not think that there were problems with our finding of significant differences. Some of the results should be investigated further in the future. Third, we did not have data on biochemistry levels in blood, participants’ lack of motivation or increase in comorbidities.

## 5. Conclusions

Discontinuation of CR influenced standing balance and limb muscle mass. Restart of CR may quickly improve a patient’s balance, but the recovery of limb muscle mass might take more time or additional daily physical activities. The recent pandemic-related interruption of CR should inspire the development of alternatives that could ensure the continuity of CR in future crises.

## Figures and Tables

**Table 1:** Patient characteristics before restart CR.

Variables	*n* = 78
Age, years	74.1 ± 8.5
Male, *n* (%)	49 (62.8)
Smoking, *n* (%)	6 (9.1)
Hypertension, *n* (%)	67 (85.9)
Diabetes mellitus, *n* (%)	37 (47.4)
Dyslipidemia, *n* (%)	66 (84.6)
Chronic kidney disease, *n* (%)	46 (59.0)
COPD, *n* (%)	2 (2.6)
Ischemic heart disease, *n* (%)	45 (57.7)
Heart failure, *n* (%)	40 (51.3)
Cardiomyopathy, *n* (%)	8 (10.3)
Macrovascular disease, *n* (%)	12 (15.4)
Peripheral artery disease, *n* (%)	8 (10.3)
BNP, pg/mL	79.7 (49–198)
LVEF, %	60.9 ± 12.5

CR, cardiac rehabilitation, COPD: chronic obstructive pulmonary disease, BNP: brain natriuretic peptide, LVEF: left ventricular ejection fraction.

**Table 2:** The changes of FFQ before DC, at restart CR and after 3 months.

	Before DC	Restart CR	After 3 Months	*p* Value	*p* Value	*p* Value
	*n* = 10	*n* = 74	*n* = 63	Before DC vs. Restart CR	Restart CR vs. after 3 months	Before DC vs. after 3 months
Total calorie intake, kcal	1775 ± 323	1769 ± 436	1735 ± 362	0.49	0.11	0.11
Protein calorie, %	14.7 ± 2.6	14.4 (13.3–15.9)	14.6 (13.2–15.7)	0.26	0.99	0.24
Fat calorie, %	28.6 ± 5.5	29.8 ± 5.1	30.6 ± 4.5	0.48	0.29	0.85
Carbohydrate calorie, %	56.7 ± 7.0	55.7 ± 5.9	54.8 ± 5.4	0.99	0.37	0.76
Salt intake, g	8.77 ± 3.0	9.35 (7.0–12.0)	9.3 (7.1–11.4)	0.83	0.53	0.70
Calorie expenditure, kcal	2095 ± 489	2225 ± 564	2049 (1813–2541)	0.59	0.36	0.55
due to exercise, kcal	0 (0–138)	39 (0–92)	55 (0–150)	0.95	0.14	0.69
due to active living activities, kcal	315 ± 248	343 (155–728)	380 (192–606)	0.32	0.67	0.35
due to normal living activities, kcal	225 ± 97	284 (210–428)	293 ± 117	0.28	0.08	0.50
due to rest living activities, kcal	1441 ± 180	1340 ± 183	1341 ± 183	0.16	0.55	0.58

FFQ: frequency questionnaire, DC: discontinuation, CR: cardiac rehabilitation.^4^.

**Table 3:** Changes in body composition, before DC, at restart of CR and after 3 months.

	Before DC	Restart CR	After 3 Months	*p* Value	*p* Value	*p* Value
	*n* = 41	*n* = 67	*n* = 61	Before DC vs. Restart CR	Restart CR vs. After 3 months	Before DC vs. After 3 months
Body weight, kg	63.0 (54.3–67.5)	60.7 ± 10.8	60.2 ± 10.6	0.94	0.49	0.87
Body mass index, kg/m^2^	23.4 (22.3–25.9)	23.6 (21.9–25.6)	23.5 (21.9–25.0)	0.71	0.84	0.61
Body fat mass, kg	16.6 ± 6.4	15.8 (13.3–20.1)	15.7 (13.3–18.5)	0.63	0.24	0.25
Percent body fat, %	26.6 ± 7.6	26.9 ± 8.3	27.2 (23.2–31.1)	0.74	0.13	0.16
Body muscle mass, kg	44.5 (37.0–48.6)	41.7 ± 7.2	41.6 ± 7.2	0.41	0.25	0.20
Estimated bone mass, kg	2.50 (2.20–2.70)	2.35 (2.15–2.65)	2.35 (2.10–2.60)	<0.001	0.17	0.009
Basal metabolic rate, kcal	1240 ± 200	1215 ± 187	1208 ± 187	0.84	0.10	0.32
Trunk muscle mass, kg	24.5 (19.8–26.5)	23.2 (19.9–25.7)	23.5 (20.4–26.3)	0.65	0.60	0.94
Trunk fat mass, kg	9.83 ± 3.97	9.30 (7.85–11.8)	9.85 (7.90–11.30)	0.63	0.06	0.12

DC: discontinuation, CR: cardiac rehabilitation.

## Data Availability

The data that support the findings of this study are available from the corresponding author upon reasonable request.
